# Curcumin Is an Iconic Ligand for Detecting Environmental Pollutants

**DOI:** 10.1155/2022/9248988

**Published:** 2022-03-27

**Authors:** T. Devasena, N. Balasubramanian, Natarajan Muninathan, Kuppusamy Baskaran, Shani T. John

**Affiliations:** ^1^Centre for Nanoscience and Technology, Anna University, Chennai 608002, Tamil Nadu, India; ^2^Department of Electrochemical Engineering Laboratory, Anna University, Chennai 608002, Tamil Nadu, India; ^3^Central Research Laboratory, Meenakshi Medical College Hospital and Research Institute, Meenakshi Academy of Higher Education and Research, Kanchipuram, Tamil Nadu, India; ^4^Department of Biology, School of Natural Science, Madawalabu University, Post Box No. 247,Oromiya Region, Bale Robe, Ethiopia

## Abstract

The rapid increase in industrial revolution and the consequent environmental contamination demands continuous monitoring and sensitive detection of the pollutants. Nanomaterial-based sensing system has proved to be proficient in sensing environmental pollutants. The development of novel ligands for enhancing the sensing efficiency of nanomaterials has always been a challenge. However, the amendment of nanostructure with molecular ligand increases the sensitivity, selectivity, and analytical performance of the resulting novel sensing platform. Organic ligands are capable of increasing the adsorption efficacy, optical properties, and electrochemical properties of nanomaterials by reducing or splitting of band gap. Curcumin (diferuloylmethane) is a natural organic ligand that exhibits inherent fluorescence and electrocatalytic property. Due to keto-enol tautomerism, it is capable of giving sensitive signals such as fluorescence, luminescence, ultraviolet absorption shifts, and electrochemical data. Curcumin probes were also reported to give enhanced meterological performances, such as low detection limit, repeatability, reproducibility, high selectivity, and high storage stability when used with nanosystem. Therefore, research on curcumin-modified nanomaterials in the detection of environmental pollution needs a special focus for prototype and product development to enable practical use. Hence, this article reviews the role of curcumin as a natural fluorophore in optical and electrochemical sensing of environmentally significant pollutants. This review clearly shows that curcumin is an ideal candidate for developing and validating nanomaterials-based sensors for the detection of environmental pollutants such as arsenic, lead, mercury, boron, cyanide, fluoride, nitrophenol, trinitrotoluene, and picric acid and toxic gases such as ammonia and hydrogen chloride. This review will afford references for future studies and enable researchers to translate the lab concepts into industrial products.

## 1. Introduction

The alarming situation of environmental pollution has increased the demand for novel nanomaterials for the ultrasensitive detection of pollutants. Fabrication of specific and sensitive nanoplatform for the detection of various environmental pollutants such as metal ions, anions, and explosives has become essential to solve many environmental issues and their adverse biological effects [1, 2]. Ligand-functionalised nanostructures exhibit novel behavior and enhanced functional ability. Organic ligands are capable of increasing the adsorption efficacy, optical properties, and electrochemical properties of nanomaterials by reducing or splitting of band gap [3, 4]. Among many methods, fluorescent techniques have become customary due to the simplicity of operation and ability to detect trace concentrations of pollutants. Therefore, several reports have been published on the application of fluorescent organic probe for the detection of metal ions and for anions, such as cyanide [5–9]. Advantages of fluorescent organic probes are flexibility in the synthesis protocol, and optical signals, that is, aggregation-induced enhanced emission [10, 11]. Organic fluorophores can conveniently be prepared by methods like emulsification evaporation, emulsification diffusion, solvent displacement, self-assembly, and reprecipitation methods [12, 13].

Curcumin (C_21_H_20_O_6_), a natural fluorophore is an ideal organic ligand, capable of showing alterations in their optical as well as electrochemical properties in the presence of significant environmental pollutants. Such signals can be used for the effective detection of select pollutants [14, 15]. Furthermore, the tautomeric form of curcumin is used as an electrochemical transducer and ion receptor. It is capable of offering potentiometric response with good linear ranges for toxic ions such as cyanide ions and mercuric ions. For example, chelation of mercury(II) ions and cyanide ions to curcumin decorated on graphene-modified glassy carbon electrodes (GCE) induces anodic shift and cathodic shift, respectively. As curcumin exists in keto form and enol form, it is capable of simultaneous detection of cationic and anionic pollutant in a given sample. Keto form is responsible for giving signals pertaining to cationic detection while the enol form gives signal corresponding to anionic detection. Curcumin probes were also reported to give enhanced meterological performances such as low detection limit, repeatability, reproducibility, high selectivity, and high storage stability when used with nanosystem [15]. Considering the merits of curcumin in generating optical and electrochemical signals, research work on surface functionalisation of nanomaterials with curcumin needs a special focus. Hence, this article reviews the role of curcumin as a natural fluorophore in optical and electrochemical sensing of environmentally significant pollutants such as heavy metal ions, nitrophenols and explosives such as trinitrotoluene (TNT). This review will probably afford references for future studies and enable researchers to translate the lab concepts into products and devices.

## 2. Curcumin

**Figure 1:**
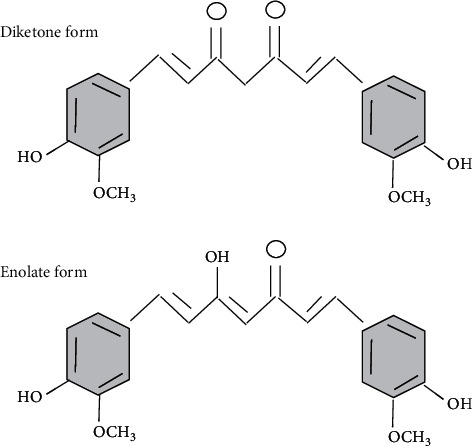
Structure of curcumin.

Curcumin [1,7-bis(4-hydroxy-3methoxyphenyl)-1,6-heptadiene-3,5-dione] is a low-molecular-weight natural phytochemical and the active principle of turmeric. Structurally, it is a diferuloylmethane containing two *o*-methoxy phenolic groups linked to the *α*, *β*-unsaturated *β*-diketone (heptadiene-dione) moiety (Figure 1). Curcumin possesses photophysical and inherent fluorescent properties. Its fluorescent properties get altered when it binds to environmental pollutants [16–21]. Hence, it is exploited as a natural fluorophore and as an electrochemical transducer to enhance the performance of nanosensors for detecting environmentally significant molecules, ions, metals, and pollutants [20–27]. It is also used to chelate metal ions and this property has been used in synthesis of functionalized nanoparticles [28–35].

It is easy to identify the binding of curcumin to nanomaterials and to pollutants by spectroscopically analyzing the band of its functional groups such as keto group, enol group, phenol group, and methoxy group. The *β*-diketone group in the central 7-carbon chain of the curcumin has a high driving force to chelate with metal ions [36]. In alkaline pH, the enolic form of curcumin predominates so that the heptadienone chain functions as an electron rich site/electron donor [37]. This property is responsible for bonding with electron deficient analytes such as explosives and capable of sensing them with high sensitivity [20]. Nanomaterial Surface Energy Transfer (NSET) was found to be more efficient when curcumin is used as a ligand and the resultant optical signal is used to detect pollutants [20]. Zinc curcumin oxide nanoparticles, coated with chitosan (Zn(*Cur*)O-Chi), were found to be an excellent nanoprobe capable of giving fluorescence signal [38].

The enol band becomes more clear and prominent and broadness increases when curcumin forms a hybrid with nanomaterial/metal ions as compared to free curcumin as evidenced by Fourier Transform Infrared Spectroscopic results [17]. The hydroxyl and methoxy groups on the phenyl rings of curcumin are electron-donating systems expected to even cause stronger hydrogen bond effect. So, any weakening of these groups' electronegativity (phenyl ring and/or the existing methoxy and hydroxy groups on it) by forming bonds or conjugating with other moieties would cause the hydrogen bond strength to decrease, thus allowing the *enol* peak to appear more clearly [17]. Curcumin is capable of showing a red shift in the UV absorption peak after interacting with metal ions or nanostructures. This is attributed to a decrease in band gap between Π − Π^*∗*^ electronic transition of curcumin due to their interaction [17]. Energy gap for curcumin decreases on binding with divalent cations leading to a shift in UV absorbance peak to longer wavelength. Patra et al. have reported the role of curcumin in a fluorimetric detection system. Curcumin was encapsulated in nanoparticle-assembled microcapsules prepared from poly[diallylammonium chloride-co-(sulfur dioxide)] [26].

In addition to its role in enhancing the optical (spectroscopic, colorimetric, and fluorescence) signals, curcumin is also capable of enhancing the electrochemical signals as evidenced by cyclic voltammetry (CV) and differential pulse voltammetry (DPV) [20, 39]. The methylene radical at the central 7-carbon chain of curcumin has crucial involvement in its redox behavior due to H-shift from the methylene group [40]. Masek et al. have revealed the optical and electrochemical characteristics of curcumin that are applicable to sensing pollutant analytes [41]. Metal organic framework (MOF) exhibits electrochemical activity and it is a potential material for electrochemical sensors [42–44]. Dang et al. have adsorbed curcumin into nano-MOF and suggested that the conjugate can emerge as a promising sensing platform generating quality electrochemical signals [45]. Mars et al. have reported the dual functionality of curcumin as a fluorescence and electrochemical transducer in a nanoplatform formed by curcumin and graphene sheets [46]. Integration of electrochemical techniques with nanoconjugates for sensor applications results in enhanced sensitivity, better limit of detection, and robustness of the sensors [47]. Above all, curcumin is environment-friendly and can easily be biodegraded on disposal. Hence, curcumin is an ideal candidate for developing and validating nanomaterials-based sensors for the detection of environmental pollutants such as metal ions, anions such as cyanide, fluoride, explosives such as TNT, and toxic gases.

## 3. Heavy Metals

### 3.1. Arsenic

Natural sources of arsenic include volcanic ash, mineral crust, mineralized ground water, and atmospheric dusts. Arsenic pollution in water has become an alarming environmental issue as the US Environmental Protection Agency (EPA) has reduced the maximal contamination level (MCL) of arsenic in drinking water from 50 *µ*g/L to 10 *µ*g/L (0.01 ppm) [48]. Furthermore, arsenic contamination causes deleterious effects in vital organs [49–51] and also affects the mental development in children [52]. Curcumin establishes electron-hole recombination [53] when doped into the defects of ZnO (forming CM-ZnO). As the defects are the spots of luminescence, it could be suggested that curcumin plays a dual role of (i) quenching the visible photo luminescence (visible emission) at 560 nm and (ii) the enhancement of UV emission (at 358 nm) as compared to free ZnO. According to Moussawi and Patra, at an excitation wavelength of 425 nm, the curcumin-doped ZnO is sensitive to arsenic. The detectable concentration range is around 100 to 3000 ppb. During the sensing process, the arsenic adsorbs to the CM-ZnO by pseudo-second-order model with excellent adsorption rates as compared to ZnO [17]. The arsenic is capable of binding to the phenolic ring and/or the hydroxyl group of the curcumin to induce a change in fluorescence and hence, curcumin can also contribute to the water treatment by the removal of arsenic. Overall, CM-ZnO's higher affinity to arsenic is responsible for the sensing and water treatment efficacy.

Sirawatcharin et al. have developed a method for UV-visible photometry-based detection and naked-eye detection of arsenic in water using difluoroboron-curcumin (BF2-curcumin). BF2-curcumin was prepared using borontrifluoride diethyletherate ((C2H5)2OBF3), and curcumin was an orange solution, which showed a maximum absorbance at 509 nm. In the presence of arsenic, the absorbance maxima were shifted to 632 nm. The red shift was accompanied by the change of the solution color from orange to blue with the detection limit of 25 *μ*M. Additionally, BF2-curcumin solution was coated to resin and used as an equally effective naked-eye sensing system with the detection limit of 30 *μ*M [54].

### 3.2. Lead

According to World Health Organization (WHO), the maximum permissible level of Pb^2+^ is 0.05 mg/L while excessive levels of Pb^2+^ lead to environmental and biological toxicity. Biological effects include hypertension and functional impairment of vital organs such as heart, liver, brain, and kidney. To be specific, it affects mental coordination, learning abilities, and IQ in children [55–58]. Atomic absorption spectrometry, inductively coupled plasma-mass spectrometry, anodic stripping voltammetry and reversed-phase high-performance liquid chromatography, and UV-visible spectroscopy are conventional techniques used for lead detection [59–62]. However, these techniques are time-consuming and require skilled man power, and equipments. But colorimetric technique together with optically tunable nanomaterial probe is ideal, highly specific, and ultrasensitive towards the detection of lead. Daniel et al. have developed a nanofiber-based cheap, disposable sensing strip with curcumin for the rapid detection of lead. The strip was fabricated using curcumin-loaded cellulose acetate nanofibers (approximately 100 nm) for the selective detection of lead amidst other heavy metal cations. The strips were capable of showing visible color change from yellow to orange-brown in the presence of lead ion detection. On complexing with lead ions, FTIR peaks of curcumin corresponding to OH group and carboxyl moiety were reported to be shifted, suggesting the formation of direct bonding or intermolecular hydrogen boding through OH and C=O groups [63].

### 3.3. Mercury

Mercury enters into terrestrial and aquatic ecosystem from various sources like coal, metal ores, paint, electronic devices, chlor-alkali plants, and catalyst [64–67]. Environmental contamination with mercury ions leads to bioaccumulation in fishes, which are the vital components of the food chain. Human consumption of marine and shell fishes are important exposure factor [19]. Therefore, mercury contamination is a main concern for humans. DNA breakage [68], carcinogenesis [69], and damage to vital organs like brain, heart, kidney, and digestive system [70, 71] are the major toxic effects of mercuric ions. According to the WHO, the permissible level of Hg^2+^ ions in drinking water is 0.006 *µ*g/ml. Curcumin was adhered to a MnO_2_-graphene-modified GCE to enhance the performance of the potentiometric detection of mercury(II) ions. Anodic shift was obtained in the presence of mercuric ions with a peak potential at 0.82 V with the detection limit of 19.2 nM [15]. Chronoamperometry and differential pulse voltametry (DPV) confirms the curcumin-based detection of Hg(II) ions. Once the Hg(II) binds to curcumin-loaded nanosystem, there was a shift in anodic potential causing a new oxidation peak of curcumin-mercury complex at 0.82 V as revealed by DPV studies. The C=O group in the keto form of curcumin was thought to generate the signals on sensing mercury(II) ions. Delocalisation of electronic charge form the chelator site towards the metal center was responsible for the new anodic shift caused by the curcumin-metal complex. It should be noted that the free curcumin easily oxidizes as compared to the cation-curcumin complex [15].

Pourreza et al. have developed a nanocurcumin-based paper strip sensor for detecting mercury in water samples [72]. The sensor prepared using wax dipping technique was highly selective and portable. The color intensity was the signal produced in the sensor, which showed a linear increase with an increase in the concentration of Hg^2+^ ions. This team has optimized various parameters such as pH, buffer, ionic strength, the amount of curcumin nanoparticles, and the target ions. A linear range of 0.5–20 *μ*g mL^−1^ of Hg^2+^ with the limit of detection of 0.17 *μ*g mL^−1^ (in absence of preconcentration). After 50 times preconcentration, the linear range was 0.01–0.4 *μ*g mL^−1^ of Hg^2+^ with the limit of detection of 0.003 *μ*g mL^−1^ [72].

Atomic absorption spectroscopy (AAS), atomic fluorescence spectrometry (AFS), cold vapour-AFS (CV-AFS), inductively coupled plasma-mass spectroscopy (ICP-MS), ICP-isotope dilution mass spectrometric (ICP-IDMS), and microwave-induced plasma atomic emission spectroscopy (MIP-AES) are used for the detection of mercury. Nevertheless, colorimetry method is capable of generating signals detectable by naked eye and it is also cheap and feasible [73]. Functionalised gold nanoparticles are good detection candidates for colorimetric sensing of many metal ions including mercury [74, 75]. We have green synthesized curcumin analog functionalized gold nanocubes and confirmed their interaction [30]. Kumar et al. [76] have synthesized curcumin-capped gold nanoparticles for the colorimetric detection of mercury in aqueous solution. The color of curcumin-capped gold nanoparticles was wine red which changed to blue in the presence of Hg^2+^ ions. Mercury detection (binding of Hg to curcumin) was also accompanied by a decrease in the absorbance at around 523 nm. The detection of Hg^2+^ was linear from 2 *µ*M to 10 *µ*M and the reported detection limit was 2 *µ*M. The acetylacetone group of curcumin was responsible for binding to the mercury ion [76].

### 3.4. Boron

Boron enters into the environment due to weathering or from consumer products, such as cosmetics and laundry products. Boron enters into food chains from the plants that absorb boron. Excessive boron absorbed by animals or humans due to the consumption of boron accumulated in plants results in reproductive toxicity. Boonkanon et al. [77] have doped curcumin nanoparticle into starch film and developed a rapid, cost effective, and ultrasensitive probe for sensing boron in waste water. As the curcumin nanoparticles were synthesized using turmeric powder and the starch was sourced from tapioca, this sensor was referred to as a green probe. The thin film of starch decorated with curcumin nanoparticles was coated onto a spoon forming a yellow color. In the presence of boron (pH 9), yellow changed to red. The sensor was reusable for 10 times and the components showed excellent biodegradability. The sensor showed a detection limit of 0.052 mg L^−1^ boron. The film can be used in conjunction with digital image colorimetry (DIC). This causes a green color layer in the reflected light image of the red-brown product and achieved high sensitivity. The sensor resisted storage for up to a year [77].

## 4. Toxic Anions

### 4.1. Sulphide

Copper is an important environmental pollutant [78] and according to WHO, the tolerable concentrations of copper in drinking water is 2 ppm [79]. Excess levels of copper ions lead to oxidative stress, resulting in childhood cirrhosis, prion disease, Menkes disease, Parkinson's disease, and Wilson disease [80]. Similarly, the sulfide (S^2-^) anions are generated due to synthesis of sulfur and sulfuric acid, dyes, and cosmetics. Toxic effects of sulfide anions include suffocation, loss of consciousness, and irritation of mucous membranes [81]. In aqueous solution, the protons interact with sulfide anions and generate HS−, which is even more toxic as it causes distress, unconsciousness, central nervous system damage, and asphyxiation [82]. Copper ions and sulfide anions compete for binding to curcumin. Hence, curcumin can be used as an “off-on” fluorescent probe for the simultaneous detection of copper ions and sulfide anions.

Curcumin nanoparticles prepared by simple precipitation method were used as an “off-on”-type fluorescence sensor for the selective detection of copper ions. The curcumin probe was responsible for the sequential recognition of cations and anions (copper and sulfide) based on the displacement approach. When the sensor detects copper ions, it leads to chelation-induced ‘“fluorescence quenching” while binding of sulfide ions recovers the quenched fluorescence. Job's, Hill's, and Benesi–Hildebrand plots were used to determine the stoichiometry and complexing capacity of curcumin nanoparticles with Cu^2+^. Carbonyl and hydroxyl groups of curcumin nanoclusters are regarded as molecular recognition sites and they have the ability to recognize and bind with metal ions [83].

### 4.2. Cyanide

Cyanide is released into the environment and water sources due to gold extraction process, plastic production, and dye production [84–87]. According to the World Health Organization, the allowable exposure level for cyanide is 1.9 mM and the lethal dose is 2.6 mM. Hence, rapid and sensitive detection of cyanide has become important to protect the health and environment. The carbonyl moiety of curcumin and its derivative are capable of nucleophilic addition with anions such as cyanide and produce a change in FTIR signals. The change in the carbonyl peak after cyanide addition was significant [14, 88]. The color of curcumin-based paper sensor changed from yellow to red in the presence of cyanide ions in water. The color intensity increased with the increase in cyanide concentration. Nucleophilic addition of cyanide to curcumin results in the deprotonation of phenolic site of the latter. This results in the conversion of benzenoid form into quinonoid form. At the same time, the cyanide solution becomes acidic (low pH) by gaining the protons generated from curcumin. Curcumin turns from yellow to red when its benzenoid form is converted into quinonoid form. Firstly, the carbonyl group of curcumin is highly susceptible to nucleophilic addition while the cyanide ions have high nucleophilicity. Secondly, cyanide functions as a base and its addition renders the curcumin enolic.

Curcumin has been used as a molecular probe for sensing cyanide in aqueous solution. A solution containing acetonitrile : water (90 : 10) was used as a medium for curcumin (yellow solution). In the presence of cyanide, curcumin solution undergoes a visual change from bright yellow to dark orange. In addition, the curcumin was capable of showing a bathochromic shift in UV spectrum in the presence of the target analyte. The absorption band was shifted from 428 nm to 520 nm. The fluorescence emission of curcumin got quenched on binding to cyanide ions. The visual change, the spectral change, and the fluorescent change were not produced by other interference monovalent ions such as fluoride, chloride, bromide, and so on. The lower detection limit was found to be 2.3 × 10^−6^ M [89].

Curcumin is used for the electrochemical detection of cyanide when functionalized to a glassy carbon electrode (GCE) modified with MnO_2_-graphene complex [15]. Potentiometric signals were obtained. Cathodic shift was obtained in the presence of cyanide with a new DPV peak potential at 0.12 V (indicative of curcumin-cyanide complex) with the detection limit of 28.3 nM (ppb). At the same time, significant decrease in the oxidation current of curcumin was obtained [15]. The signal was attributed to the presence of hydroxyl group in the enolic form of curcumin, which was capable of forming hydrogen bond with the target analyte. The cathodic potential shift caused by the cyanide binding was thought to be associated with the amplification of the electron density of the redox active electrochemical transducer, curcumin [90, 91].

### 4.3. Fluoride

Fluoride enters into the environment (especially water) in natural forms like fluorite, fluorapatite, and cryolite, or from anthropogenic sources such as coal burning, oil refining, steel production, brick-making industries, and phosphatic fertilizer plants. Majority of fluoride accumulates in water. Excessive exposure to fluoride causes fluorosis of bone and teeth [92]. Curcumin was an integral recognition probe in an optical sensor developed using upconversion nanoparticles (UCNPs). This sensor was used for the detection of fluoride ions using fluorescence and colorimetric signals. In the presence of fluoride, the absorption peak of curcumin shows a bathochromic shift and an upconversion fluorescence quenching at 546 nm and 657 nm via inner filter effects. The linear range for fluorescence and colorimetric analysis was 25–200 *μ*M and 5–200 *μ*M with the detection limits as low as 25 *μ*M (ca. 0.48 ppm) and 5 *μ*M (ca. 0.10 ppm), respectively [93].

Wu et al. have used curcumin as a colorimetric and fluorescent chemosensing probe for the selective recognition of fluoride ion. In the presence of fluoride ion, the absorption spectra of curcumin show red shift from 418 nm to 562 nm with a concomitant color change from yellow to purple. The spectral change was attributed to the deprotonation of curcumin and the formation of anionic complex between the two hydroxyl groups of curcumin and the fluoride ions. Additionally, the fluorescence of curcumin quenches on detecting fluoride ions which was reported to be due to the photon-induced electron transfer in the presence of fluoride. Job plot curve provided evidence for 1 : 1 anionic complex formation [16].

Mejri et al. have used curcumin as an electrochemical transducer for the sensitive detection of fluoride simultaneous to cyanide and mercury(II) ions. A glassy carbon electrode (GCE) modified with MnO_2_-graphene complex was used as a working electrode. The electrode nanohybrid on the GCE surface was decorated with curcumin and the potentiometric signals were recorded. Sensing of fluoride was confirmed by a cathodic shift and a decrease in the oxidation current of curcumin by 2.11 *µ*A, which was attributed to the hydroxyl group in the enolic form of curcumin. Sensing of fluoride was characterized by new DPV peak potential of −0.24 V (characteristic of curcumin-fluoride complex) with the detection limit of 17.2 nM [15]. The cathodic potential shift caused by the fluoride binding was thought to be associated with the amplification of the electron density of the electrochemical transducer, curcumin [90, 91].

## 5. Nitrogen Derivatives

### 5.1. Trinitrotoluene (TNT)

Trinitrotoluene (TNT) is a dangerous chemical weapon and an explosive material. This emphasizes the importance of homeland security, battlefield protection, and industrial and environmental security. Therefore, the detection of even trace amount of TNT has become very essential and is emerging as an important area of research in the field of environmental sensors [94]. Commonly used methods like ion mobility spectrometry, mass spectrometry, and gas chromatography are laborious, show less sensitivity, and are not capable of on-site detection. Nanomaterial Surface Energy Transfer (NSET) is superior to Fluorescence Resonance Energy Transfer (FRET) in efficiently transferring the energy from a donor molecule to the surface of a nanomaterial covering a distance twice that of FRET [95, 96].

Pandya et al. [20] have used nanocurcumin probe and developed an aggregation technique for detecting trinitrotoluene (TNT). This technique involves NSET between the electron-rich moiety of nanocurcumin and the electron deficient TNT. This probe was highly selective over other explosive compounds and showed remarkable sensitivity, detecting a concentration of 1 nM TNT. The detection system showed several-fold fluorescence enhancement. In alkaline pH, the enol form of curcumin predominates so that the heptadienone chain functions as an electron rich site/electron donor [37]. A *π*-donor-acceptor interaction between electron-deficient TNT and the *π*-electron-rich curcumin nanoparticles takes place, which is responsible for forming aggregates. As a result of aggregation, the yellow curcumin changes to orange and then to red. Color change was accompanied by a substantial bathochromic shift in the UV spectral band [37]. The degree of red shift and the intensity of the orange color and the red color of the solution depend on the size of the aggregate, which in turn is proportional to the concentration of the TNT.

Curcumin-silver nanoparticles conjugate interact with TNT via p-donor-acceptor interaction with excellent selectivity. Curcumin functionalized with PVP-capped Ag NPs can be used as an ultrasensitive optical probe for TNT detection. Curcumin-silver conjugate functions as a UV-visible spectroscopy, DLS, and Surface-Enhanced Raman spectroscopy- (SERS-) based multiple tier real-time probe. This probe showed an ultrasensitive detection of TNT up to 0.1 nM level. This technique has an added advantage of producing visible flakes in the presence of TNT, thus enabling naked eye detection [97].

### 5.2. Picric Acid

Picric acid (2,4,6-trinitrophenol) is a nitro-aromatic explosive, mainly used in rocket fuel, fireworks, and matches [98, 99]. It is also used in the preparation of dye and in pharmaceutical and in leather industries [100]. Hazardous industrial wastes containing picric acid are not only hazardous to environment but also deleterious to the biological systems [101]. MOFs [102], semiconductor quantum dots [103, 104], and fluorophore-nanomaterial hybrids [105] are used for the detection of picric acid. These methods, however, suffer from the demerit of interference by structural analogs and toxicity of the materials used [37]. Amolins et al. have prepared a co-polymeric microsphere-curcumin hybrid by one-pot precipitation method [37]. Hexa-chlorocyclotriphosphazene (HCCP) was the polymeric microsphere that contains electron-rich nitrogen. HCCP exhibited affinity towards the proton-rich picric acid, thereby quenching the fluorescence of the curcumin. The sensor was selective over other interfering molecules such as nitrotoluene, nitrobenzene, and so on and showed a sensitivity of 85 ppb. Gogoi and Sen Sarma have developed a cost-effective detection system based on curcumin-cysteine and curcumin-tryptophan for the selective detection of picric acid in aqueous media. The amino acid conjugates of curcumin establish electrostatic bonding with the picric acid and result in aggregation. The aggregates are responsible for the fluorescence enhancement by nearly 25-fold. This sensing system shows a detection limit of 13 nM of picric acid with low interference by other analogues. Amino acid-curcumin conjugates have the potential to sense picric acid in real environmental samples [106].

Environmentally benign greener sensing approach is very significant in perspective of health and environmental safety and homeland security. In this context, Chakravarty et al. [107] developed a new greener method for the sensing of picric acid by using the biomaterials scutellarin-hispiduloside and curcumin modified with the green solvent glycerol. The sensing was due to the fluorescence quenching, governed by the FRET between the fluorophore and the quencher. This sensing system was selective towards picric acid amidst other structurally similar nitro-aromatic compounds [107].

### 5.3. Nitrophenol

Nitrophenols are regarded as important environmental pollutant as they contaminate water bodies due to improper disposal of its sources like pesticides, paints, dyes, plastics, and rubber products [108]. Musilová et al. [109] have suggested 7 × 10^−8^ mol/L as the restricted concentration of nitrophenol in water. Higher concentrations of nitrophenols in water can induce headache, drowsiness, and nausea [110]. Anchoring of curcumin to chitosan film establishes a hydrogen between them. This could be due to interaction between the oxygen of hydroxyl group on the benzene ring of curcumin with either the hydroxyl or the amino group of the chitosan [111]. The resulting hybrid film was the apt sensor for ortho nitrophenol (ONP) as well as fluoride ion. Serial increase in the concentrations of curcumin resulted in an increase in the quantity of bound curcumin per gram of chitosan. But beyond a certain limit (i.e., above 20.0 × 10^−4^ g/mL), there was no increase showing the saturation point. ONP interacts with the curcumin forming hydrogen bond that is not fluorescent active. Also, by using the Stern–Volmer equation, the quantitative detection of the targets is possible. Thus, the fluorescence quenching of curcumin enables both qualitative and quantitative detection of the ONP and fluoride ions as well. Interestingly, the limit of detection level for ONP and fluoride ion provided by curcumin was well lower than the values given by other methods like UV spectroscopy and chromatography [106]. An electrochemical sensor was developed by modifying gold electrode with curcumin-amino acid conjugate monolayer and further functionalisation with confined copper nanospheres. This sensor showed excellent sensitivity and selectivity for the detection of *p*-nitrophenol. The copper nanosphere was reported to enhance electron transfer (less resistance) between the probe and electrode and contribute to sensing of the target. The sensor showed a practical feasibility for detecting the target in real environmental media. Furthermore, the sensing platform is cost-effective, reproducible, and easy to fabricate [112].

### 5.4. Hydrazine

Hydrazine and its derivatives are used as reducing agents in fuel cells, and in insecticides, explosives, rocket propellants, metal film manufactures, photographic chemicals, plastic blowing agents, and as oxygen scavengers in boilers [113, 114]. Hydrazine easily penetrates through skin; it affects blood production and it is also carcinogenic, hepatotoxic, and nephrotoxic [115]. Therefore, hydrazine detection is of utmost importance. Zheng and Song [21] have developed a curcumin-MWCNT modified glassy carbon electrode by electrode position. The electrochemical sensor was used for the amperometric detection of hydrazine. The sensing system showed an electrocatalytic activity towards the oxidation of hydrazine at a reduced overpotential accompanied by an increased peak current as compared to unmodified electrode. This sensor has the advantages of easy fabrication, speedy protocol, high sensitivity, and good reproducibility for hydrazine determination. The linear detection range was 2–44 *µ*M, while the detection limit was 1.4 *µ*M. The two *o*-methoxy phenolic groups in curcumin molecule functions as an electrocatalytic moiety for hydrazine oxidation.

### 5.5. Pyridine and Pyrrole

2-Vinyl pyridine (2-VP) is used as a precursor used in the synthesis of special polymers such as latex, styrene, butadiene, and so on. In medical field, it is used in the synthesis of veterinary anthelmintic and a pharmaceutical called axitinib. The toxic nature of 2-VP in spite of its extensive application in the laboratories emphasizes the significance of its sensitive detection. As per the Material Safety Data Sheet (MSDS), 2-VP is flammable, and it produces allergic/corrosive in skin, eyes, and respiratory system [116]. A novel composite of polyglycerol acrylate and curcumin has been used for the detection of 2-vinyl pyridine by dual mode (i.e., by fluorescence and by electrical properties). In the presence of 2-VP, the fluorescence of the composite quenches. The electrical properties such as DC current-voltage characteristics and AC impedance response change in the presence of saturated vapor of 2-VP. In the presence of the target vapor, the current density decreases up to 84.7 and 83.13% at an applied bias voltage of −5 to +5 V, respectively. The impedance hikes up to about 79% at lower frequency range [117].

Pyrrole is an intermediate chemical in the manufacture of dyes, herbicides, perfumes, drug manufacturing and a cross-linking agent for resins. Intraperitoneal, oral, and subcutaneous administration of pyrrole was reported to induce toxicity in experimental animals as well as in aquatic organisms. Therefore, pyrrole is an important pollutant to be detected in the environment. Curcumin organogelator was prepared using N′1,N′6-bis(3-(1-pyrrolyl)propanoyl) hexanedihydrazide (DPH). This compound exhibited dual advantages of structural similarity with pyrrole and a remarkable enhancement in photoluminescence. Hence, the curcumin organogel was proved to be an optical sensing platform for the detection of pyrrole [118].

## 6. Toxic Gases

Amshel et al. have reviewed about ammonia toxicity [119]. Leakage of anhydrous ammonia gas or vapor of liquor ammonia from the site of production, storage, or transportation increases the risk for inhalational and dermal exposure [120, 121]. Ammonia-based fertilizers and ammonia produced by decaying manure increase the risk for inhalational exposure [122, 123]. Acute exposure to ammonia causes acute pulmonary congestion, edema, and desquamation of the bronchial epithelium [124]. Curcumin was incorporated into cellulose acetate nanofiber and used for the chromatic sensing of HCl and NH_3_. Though the color of curcumin is yellow, the color of curcumin-incorporated cellulose fiber in alkaline medium was reddish brown due to the conversion of keto form into a mixed state of enol and enolate forms. Reddish brown changes to yellow in the presence of HCl which indicated the formation of keto form. Subsequent exposure to ammonia led to the recovery of reddish brown in a few seconds, indicating the reversible nature of the tautomerism. This color changing process was due to reversible change in the conjugation state of the curcumin via keto-enol tautomerism with protonation and deprotonation reactions by HCl and NH_3_ vapors [125].

Environment is exposed to hypochlorite (ClO^−^) and its protonated form (HClO/hypochlorous acid). For example, they are the major components of disinfectant, microbial agent, and bleaching agents [126]. However, higher concentrations of hypochlorite can cause deleterious effects such as tissue damage and diseases such as atherosclerosis, arthritis, and cancers [127, 128]. Yue et al. have used curcumin for the sensitive detection of hypochlorous acid. The oxygenation of *o*-methoxyphenol into a quinone form by the hypochlorous acid was reported to be responsible for the detection. Quinone form resulted in a nonfluorescent derivative of curcumin, signaling the presence of hypochlorite. This probe showed a high sensitivity for hypochlorous acid as very low detection limit 0.065 *μ*M [129].

## 7. Conclusion

Rapid increase in the industrial revolution and automobile exhaust is environmentally hazardous. Incessant monitoring of the pollutants is essential to prevent environmental deterioration. So far, there is no review on the detection and quantification of heavy metals, toxic anions, and explosives using curcumin-based nanoprobes. Therefore, we focused on reviewing curcumin-based sensors that are competent in sensing heavy metals, anions, and explosives as summarized in Table 1. We discussed about the specificity, sensitivity, and analytical performance of the detection system. This review clearly reveals that curcumin-based nanosensors are ideal and competent in sensing environmental pollutants by optical and electrochemical methods. Therefore, this review will be of major interest for researchers, scientists, and industrialists in order to upgrade these sensors from lab to industrial level.

## Figures and Tables

**Table 1:** Summary of curcumin-based electrochemical and optical sensing platform for detecting environmental pollutants.

S. no.	Sensing element	Target analyte	Detection method
1	Curcumin-doped zinc oxide nanoparticles	Arsenic	Fluorescence
2	Curcumin-difluoroboron	Arsenic	UV-visible spectroscopy
			Colorimetry
3	Curcumin-doped cellulose acetate strip	Lead	Naked-eye detection
4	Curcumin-manganese dioxide-graphene	Mercury	Chronoamperometry
			Differential pulse voltammetry
5	Nanocurcumin	Mercury	Colorimetry
6	Curcumin-doped starch thin film	Boron	Colorimetry
7	Curcumin nanoparticles	Sulphide	Fluorescence
8	Curcumin paper strip	Cyanide	Fluorescence
9	Curcumin-manganese dioxide-graphene	Cyanide	Chronoamperometry
			Differential pulse voltammetry
10	Curcumin-UCNPs	Fluoride	Fluorescence
			Colorimetry
11	Curcumin-manganese dioxide-graphene	Fluoride	Chronoamperometry
			Differential pulse voltammetry
12	Curcumin nanoparticles	Trinitrotoluene	UV-visible spectroscopy
			Colorimetry
13	Curcumin-silver nanoparticles	Trinitrotoluene	UV-visible spectroscopy
			Surface-enhanced Raman spectroscopy
			Dynamic light scattering
14	Curcumin-functionalized polymeric microspheres	Picric acid	Fluorescence
15	Curcumin-cysteine	Picric acid	Fluorescence
16	Curcumin-tryptophan	Picric acid	Fluorescence
17	Curcumin-glycerol	Picric acid	Luminescence
18	Curcumin-doped chitosan thin film	Nitrophenol	Fluorescence
19	Curcumin-amino acid-copper nanospheres	Nitrophenol	Cyclic voltammetry
			Electrochemical impedance spectroscopy
20	Curcumin-functionalized on MWCNTs	Hydrazine	Amperometry
21	Curcumin-functionalized polyglycerol acrylate	2-Vinyl pyridine	Fluorescence
			Impedence
22	Curcumin organogelator	Pyrrole	Photoluminescence

## Data Availability

The data used to support the finding of this study are included within the article.

## References

[1] Ma B., Wu S., Zeng F., Luo Y., Zhao J., Tong Z. (2010). Nanosized diblock copolymer micelles as a scaffold for constructing a ratiometric fluorescent sensor for metal ion detection in aqueous media. *Nanotechnology*.

[2] Hou X., Zeng F., Du F., Wu S. (2013). Carbon-dot-based fluorescent turn-on sensor for selectively detecting sulfide anions in totally aqueous media and imaging inside live cells. *Nanotechnology*.

[3] Qiu R., Zhang D., Mo Y. (2008). Photocatalytic activity of polymer-modified ZnO under visible light irradiation. *Journal of Hazardous Materials*.

[4] Liao S.-H., Jhuo H.-J., Cheng Y.-S., Chen S.-A. (2013). Fullerene derivative-doped zinc oxide nanofilm as the cathode of inverted polymer solar cells with low-bandgap polymer (PTB7-Th) for high performance. *Advanced Materials*.

[5] Qi X., Jun E. J., Xu L. (2006). New BODIPY derivatives as OFF−ON fluorescent chemosensor and fluorescent chemodosimeter for Cu2+: cooperative selectivity enhancement toward Cu2+. *Journal of Organic Chemistry*.

[6] Zhou Y., Wang F., Kim Y., Kim S.-J., Yoon J. (2009). Cu2+-Selective ratiometric and “Off-On” sensor based on the rhodamine derivative bearing pyrene group. *Organic Letters*.

[7] Li X., Zhu Q., Tong S., Wang W., Song W. (2009). Self-assembled microstructure of carbon nanotubes for enzymeless glucose sensor. *Sensors and Actuators B: Chemical*.

[8] Singh A., Raj T., Aree T., Singh N. (2013). Fluorescent organic nanoparticles of biginelli-based molecules: recognition of Hg2+ and Cl- in an aqueous medium. *Inorganic Chemistry*.

[9] Bhopate D. P., Kolekar G. B., Garadkar K. M., Patil S. R. (2013). Cetyltrimethylammonium bromide stabilized perylene nanoparticles for fluorimetric estimation of bicarbonate (HCO3−) anion: spectroscopic approach. *Analytical Methods*.

[10] Mahajan P. G., Desai N. K., Dalavi D. K., Bhopate D. P., Kolekar G. B., Patil S. R. (2015). Cetyltrimethylammonium bromide capped 9-anthraldehyde nanoparticles for selective recognition of phosphate anion in aqueous solution based on fluorescence quenching and application for analysis of chloroquine. *Journal of Fluorescence*.

[11] Bhopate D. P., Mahajan P. G., Garadkar K. M., Kolekar G. B., Patil S. R. (2014). Pyrene nanoparticles as a novel FRET probe for detection of rhodamine 6G: spectroscopic ruler for textile effluent. *RSC Advances*.

[12] Li S., He L., Xiong F., Li Y., Yang G. (2004). Enhanced fluorescent emission of organic nanoparticles of an intramolecular proton transfer compound and spontaneous formation of one-dimensional nanostructures. *The Journal of Physical Chemistry B*.

[13] Bhopate D. P., Mahajan P. G., Garadkar K. M., Kolekar G. B., Patil S. R. (2015). Polyvinyl pyrrolidone capped fluorescent anthracene nanoparticles for sensing fluorescein sodium in aqueous solution and analytical application for ophthalmic samples. *Luminescence*.

[14] Paden R. R., Oracion J. P. L., De La Rosa L. B. (2021). Design and fabrication of a low-cost curcumin-based paper sensor for rapid “naked-eye” cyanide sensing. *Materials Today Proceedings*.

[15] Mejri A., Mars A., Elfil H., Hamzaoui A. H. (2018). Graphene nanosheets modified with curcumin-decorated manganese dioxide for ultrasensitive potentiometric sensing of mercury(II), fluoride and cyanide. *Mikrochimica Acta*.

[16] Wu F.-Y., Sun M.-Z., Xiang Y.-L., Wu Y.-M., Tong D.-Q. (2010). Curcumin as a colorimetric and fluorescent chemosensor for selective recognition of fluoride ion. *Journal of Luminescence*.

[17] Moussawi R. N., Patra D. (2016). Modification of nanostructured ZnO surfaces with curcumin: fluorescence-based sensing for arsenic and improving arsenic removal by ZnO. *RSC Advances*.

[18] Raj S., Shankaran D. R. (2016). Curcumin based biocompatible nanofibers for lead ion detection. *Sensors and Actuators B: Chemical*.

[19] Driscoll C. T., Mason R. P., Chan H. M., Jacob D. J., Pirrone N. (2013). Mercury as a global pollutant: sources, pathways, and effects. *Environmental Science and Technology*.

[20] Pandya A., Goswami H., Lodha A., Menon S. K. (2012). A novel nanoaggregation detection technique of TNT using selective and ultrasensitive nanocurcumin as a probe. *The Analyst*.

[21] Zheng L., Song J.-f. (2009). Curcumin multi-wall carbon nanotubes modified glassy carbon electrode and its electrocatalytic activity towards oxidation of hydrazine. *Sensors and Actuators B: Chemical*.

[22] Pang L., Zhou Y., Gao W. (2017). Curcumin-based fluorescent and colorimetric probe for detecting cysteine in living cells and zebrafish. *Industrial & Engineering Chemistry Research*.

[23] Khorasani M. Y., Langari H., Sany S. B. T., Rezayi M., Sahebkar A. (2019). The role of curcumin and its derivatives in sensory applications. *Materials science & engineering. C, Materials for biological applications*.

[24] Madhu P., Sivakumar P. (2019). Curcumin-based fluorescent chemosensor for selective and efficient detection of picric acid. *Journal of Molecular Structure*.

[25] Mouslmani M., Bouhadir K. H., Patra D. (2015). Poly (9-(2-diallylaminoethyl)adenine HCl-co-sulfur dioxide) deposited on silica nanoparticles constructs hierarchically ordered nanocapsules: curcumin conjugated nanocapsules as a novel strategy to amplify guanine selectivity among nucleobases. *Biosensors and Bioelectronics*.

[26] Patra D., Aridi R., Bouhadir K. (2013). Fluorometric sensing of DNA using curcumin encapsulated in nanoparticle-assembled microcapsules prepared from poly (diallylammonium chloride-co-sulfur dioxide). *Microchimica Acta*.

[27] Patra D., El Khoury E., Ahmadieh D., Darwish S., Tafech R. M. (2012). Effect of curcumin on liposome: curcumin as a molecular probe for monitoring interaction of ionic liquids with 1,2-Dipalmitoyl-sn-Glycero-3-Phosphocholine liposome. *Photochemistry and Photobiology*.

[28] Nambi U. G., Devasena T., Devi V. P., Francis A. P. (2015). Graphene modified with an analog of diferuloylmethane for efficient removal of chromium from industrial waste water. *Graphene*.

[29] Ganesan M., Baskar D., Francis A. P., Devasena T. (2014). Graphene oxide nanosheets with curcumin decors are effective adsorbents for the removal of chromium from tannery effluent. *Graphene*.

[30] Devi R. A., Francis A. P., Devasena T. (2014). Green-synthesized gold nanocubes functionalized with bisdemethoxycurcumin analog as an ideal anticancer candidate. *Green Processing and Synthesis*.

[31] El Khoury E., Abiad M., Kassaify Z. G., Patra D. (2015). Green synthesis of curcumin conjugated nanosilver for the applications in nucleic acid sensing and anti-bacterial activity. *Colloids and Surfaces B: Biointerfaces*.

[32] Moussawi R. N., Patra D. (2015). Synthesis of Au nanorods through prereduction with curcumin: preferential enhancement of Au nanorod formation prepared from CTAB-capped over citrate-capped Au seeds. *Journal of Physical Chemistry C*.

[33] Zhou S.-S., Xue X., Wang J.-F. (2012). Synthesis, optical properties and biological imaging of the rare earth complexes with curcumin and pyridine. *Journal of Materials Chemistry*.

[34] Ciszewski A., Milczarek G., Lewandowska B., Krutowski K. (2003). Electrocatalytic properties of electropolymerized Ni (II) curcumin complex. *Electroanalysis: An International Journal Devoted to Fundamental and Practical Aspects of Electroanalysis*.

[35] Renfrew A. K., Bryce N. S., Hambley T. W. (2013). Delivery and release of curcumin by a hypoxia-activated cobalt chaperone: a XANES and FLIM study. *Chemical Science*.

[36] Barik A., Mishra B., Kunwar A. (2007). Comparative study of copper(II)-curcumin complexes as superoxide dismutase mimics and free radical scavengers. *European Journal of Medicinal Chemistry*.

[37] Amolins M. W., Peterson L. B., Blagg B. S. J. (2009). Synthesis and evaluation of electron-rich curcumin analogues. *Bioorganic & Medicinal Chemistry*.

[38] Arab C., El Kurdi R., Patra D. (2021). Chitosan coated zinc curcumin oxide nanoparticles for the determination of ascorbic acid. *Journal of Molecular Liquids*.

[39] Devadas B., Rajkumar M., Chen S.-M. (2014). Electropolymerization of curcumin on glassy carbon electrode and its electrocatalytic application for the voltammetric determination of epinephrine and p-acetoaminophenol. *Colloids and Surfaces B: Biointerfaces*.

[40] Jha N. S., Mishra S., Jha S. K., Surolia A. (2015). Antioxidant activity and electrochemical elucidation of the enigmatic redox behavior of curcumin and its structurally modified analogues. *Electrochimica Acta*.

[41] Masek A., Chrzescijanska E., Zaborski M. (2013). Characteristics of curcumin using cyclic voltammetry, UV-vis, fluorescence and thermogravimetric analysis. *Electrochimica Acta*.

[42] Xu Y., Li Q., Xue H., Pang H. (2018). Metal-organic frameworks for direct electrochemical applications. *Coordination Chemistry Reviews*.

[43] Zhao F., Sun T., Geng F., Chen P., Gao Y. (2019). Metal-organic frameworks-based electrochemical sensors and biosensors. *International Journal of Electrochemical Science*.

[44] Horcajada P., Gref R., Baati T. (2012). Metal-organic frameworks in biomedicine. *Chemical Reviews*.

[45] Dang Y. T., Dang M.-H. D., Mai N. X. D. (2020). Room temperature synthesis of biocompatible nano Zn-MOF for the rapid and selective adsorption of curcumin. *Journal of Science: Advanced Materials and Devices*.

[46] Mars A., Hamami M., Bechnak L., Patra D., Raouafi N. (2018). Curcumin-graphene quantum dots for dual mode sensing platform: electrochemical and fluorescence detection of APOe4, responsible of Alzheimer’s disease. *Analytica Chimica Acta*.

[47] Gumpu M. B., Sethuraman S., Krishnan U. M., Rayappan J. B. B. (2015). A review on detection of heavy metal ions in water - an electrochemical approach. *Sensors and Actuators B: Chemical*.

[48] Choong T. S., Chuah T. G., Robiah Y., Koay F. G., Azni I. (2007). Arsenic toxicity, health hazards and removal techniques from water: an overview. *Desalination*.

[49] Smith A. H., Lingas E. O., Rahman M. (2000). Contamination of drinking-water by arsenic in Bangladesh: a public health emergency. *Bulletin of the World Health Organization*.

[50] Kurttio P., Pukkala E., Kahelin H., Auvinen A., Pekkanen J. (1999). Arsenic concentrations in well water and risk of bladder and kidney cancer in Finland. *Environmental Health Perspectives*.

[51] Xia Y., Liu J. (2004). An overview on chronic arsenism via drinking water in PR China. *Toxicology*.

[52] Saha K. C. (2003). Review of arsenicosis in West Bengal, India - a clinical perspective. *Critical Reviews in Environmental Science and Technology*.

[53] Xiong H.-M. (2010). Photoluminescent ZnO nanoparticles modified by polymers. *Journal of Materials Chemistry*.

[54] Sirawatcharin S., Saithongdee A., Chaicham A., Tomapatanaget B., Imyim A., Praphairaksit N. (2014). Naked-eye and colorimetric detection of arsenic(III) using difluoroboron-curcumin in aqueous and resin bead support systems. *Analytical Sciences*.

[55] Guo L., Hong S., Lin X., Xie Z., Chen G. (2008). An organically modified sol-gel membrane for detection of lead ion by using 2-hydroxy-1-naphthaldehydene-8-aminoquinoline as fluorescence probe. *Sensors and Actuators B: Chemical*.

[56] He Q., Miller E. W., Wong A. P., Chang C. J. (2006). A selective fluorescent sensor for detecting lead in living cells. *Journal of the American Chemical Society*.

[57] Zhang L., Yao Y., Shan J., Li H. (2011). Lead (II) ion detection in surface water with pM sensitivity using aza-crown-ether-modified silver nanoparticles via dynamic light scattering. *Nanotechnology*.

[58] Zhang D., Yin L., Meng Z., Yu A., Guo L., Wang H. (2014). A sensitive fluorescence anisotropy method for detection of lead (II) ion by a G-quadruplex-inducible DNA aptamer. *Analytica Chimica Acta*.

[59] Ferhan A. R., Guo L., Zhou X., Chen P., Hong S., Kim D.-H. (2013). Solid-phase colorimetric sensor based on gold nanoparticle-loaded polymer brushes: lead detection as a case study. *Analytical Chemistry*.

[60] Kim H. N., Ren W. X., Kim J. S., Yoon J. (2012). Fluorescent and colorimetric sensors for detection of lead, cadmium, and mercury ions. *Chemical Society Reviews*.

[61] Chow E., Hibbert D. B., Gooding J. J. (2005). Electrochemical detection of lead ions via the covalent attachment of human angiotensin I to mercaptopropionic acid and thioctic acid self-assembled monolayers. *Analytica Chimica Acta*.

[62] Dai D., Xu D., Cheng X., He Y. (2014). Direct imaging of single gold nanoparticle etching: sensitive detection of lead ions. *Analytical Methods*.

[63] Daniel S., Limson J. L., Dairam A., Watkins G. M., Daya S. (2004). Through metal binding, curcumin protects against lead- and cadmium-induced lipid peroxidation in rat brain homogenates and against lead-induced tissue damage in rat brain. *Journal of Inorganic Biochemistry*.

[64] Pirrone N., Keeler G. J., Nriagu J. O. (1996). Regional differences in worldwide emissions of mercury to the atmosphere. *Atmospheric Environment*.

[65] Pirrone N., Cinnirella S., Feng X. (2010). Global mercury emissions to the atmosphere from anthropogenic and natural sources. *Atmospheric Chemistry and Physics*.

[66] Pacyna J. M., Pacyna E. G., Aas W. (2009). Changes of emissions and atmospheric deposition of mercury, lead, and cadmium. *Atmospheric Environment*.

[67] Streets D. G., Zhang Q., Wu Y. (2009). Projections of global mercury emissions in 2050. *Environmental Science and Technology*.

[68] Ben-Ozer E. Y., Rosenspire A. J., McCabe M. J. (2000). Mercuric chloride damages cellular DNA by a non-apoptotic mechanism. *Mutation Research: Genetic Toxicology and Environmental Mutagenesis*.

[69] Durham T. R., Snow E. T. (2006). Metal ions and carcinogenesis. *Cancer: Cell Structures, Carcinogens and Genomic Instability*.

[70] Hoyle I., Handy R. D. (2005). Dose-dependent inorganic mercury absorption by isolated perfused intestine of rainbow trout, *Oncorhynchus mykiss*, involves both amiloride-sensitive and energy-dependent pathways. *Aquatic Toxicology*.

[71] Zalups R. K. (2000). Molecular interactions with mercury in the kidney. *Pharmacological Reviews*.

[72] Pourreza N., Golmohammadi H., Rastegarzadeh S. (2016). Highly selective and portable chemosensor for mercury determination in water samples using curcumin nanoparticles in a paper based analytical device. *RSC Advances*.

[73] Leopold K., Foulkes M., Worsfold P. (2010). Methods for the determination and speciation of mercury in natural waters-A review. *Analytica Chimica Acta*.

[74] Lin Y.-W., Huang C.-C., Chang H.-T. (2011). Gold nanoparticle probes for the detection of mercury, lead and copper ions. *The Analyst*.

[75] Lee J.-S., Han M. S., Mirkin C. A. (2007). Colorimetric detection of mercuric ion (Hg2+) in aqueous media using DNA-functionalized gold nanoparticles. *Angewandte Chemie International Edition*.

[76] Kumar P., Paul W., Sharma C. P. (2014). Curcumin stabilized gold nanoparticle-based colorimetric sensing of mercury (II). *Trends in Biomaterials and Artificial Organs*.

[77] Boonkanon C., Phatthanawiwat K., Wongniramaikul W., Choodum A. (2020). Curcumin nanoparticle doped starch thin film as a green colorimetric sensor for detection of boron. *Spectrochimica Acta Part A: Molecular and Biomolecular Spectroscopy*.

[78] Wang S., Wang X., Zhang Z., Chen L. (2015). Highly sensitive fluorescence detection of copper ion based on its catalytic oxidation to cysteine indicated by fluorescein isothiocyanate functionalized gold nanoparticles. *Colloids and Surfaces A: Physicochemical and Engineering Aspects*.

[79] Beer P. D., Gale P. A. (2001). Anion recognition and sensing: the state of the art and future perspectives. *Angewandte Chemie International Edition*.

[80] Rombel-Bryzek A., Rajfur M., zhuk O. (2017). The impact of copper ions on oxidative stress in garden cress Lepidium sativum. *Ecological Chemistry and Engineering S*.

[81] Patwardhan S. A., Abhyankar S. M. (1988). Toxic and hazardous gases. *Colourage*.

[82] Patnaik P. (2007). A comprehensive guide to the hazardous properties of chemical substances. *Chemical and Environmental Health and Safety*.

[83] Pourreza N., Golmohammadi H. (2014). Green colorimetric recognition of trace sulfide ions in water samples using curcumin nanoparticle in micelle mediated system. *Talanta*.

[84] Jaszczak E., Polkowska Ż., Narkowicz S., Namieśnik J. (2017). Cyanides in the environment-analysis-problems and challenges. *Environmental Science and Pollution Research*.

[85] Xie F., Dreisinger D. (2009). Studies on solvent extraction of copper and cyanide from waste cyanide solution. *Journal of Hazardous Materials*.

[86] Konidena R. K., Thomas K. R. J. (2014). Selective naked-eye cyanide detection in aqueous media using a carbazole-derived fluorescent dye. *RSC Advances*.

[87] Hijji Y., Al Easa H. S., AbdelRasoul M. Natural dyes in cyanide and anion sensing.

[88] Chaicham A., Kulchat S., Tumcharern G., Tuntulani T., Tomapatanaget B. (2010). Synthesis, photophysical properties, and cyanide detection in aqueous solution of BF2-curcumin dyes. *Tetrahedron*.

[89] Hijji Y. M., Elsafy A. G., Al-Easa H. S. (2019). Curcumin a colorimetric and fluorimetric cyanide probe in aqueous system and living cells. *Analytical Methods*.

[90] Beer P. D., Chen Z., Drew M. G., Pilgrim A. J. (1994). Electrochemical recognition of Group 1 and 2 metal cations by redox-active ionophores. *Inorganica Chimica Acta*.

[91] Patil S. K., Ghosh R., Kennedy P., Mobin S. M., Das D. (2016). Potential anion sensing properties by a redox and substitution series of [Ru(bpy)3−n(Hdpa)n]2+, n = 1-3; Hdpa = 2,2′-dipyridylamine: selective recognition and stoichiometric binding with cyanide and fluoride ions. *RSC Advances*.

[92] Jha S. K., Mishra V. K., Sharma D. K., Damodaran T. (2011). Fluoride in the environment and its metabolism in humans. *Reviews of Environmental Contamination & Toxicology*.

[93] Liu Y., Ouyang Q., Li H., Zhang Z., Chen Q. (2017). Development of an inner filter effects-based upconversion nanoparticles-curcumin nanosystem for the sensitive sensing of fluoride ion. *ACS Applied Materials & Interfaces*.

[94] Wang Y., La A., Ding Y., Liu Y., Lei Y. (2012). Novel signal-amplifying fluorescent nanofibers for naked-eye-based ultrasensitive detection of buried explosives and explosive vapors. *Advanced Functional Materials*.

[95] Caygill J. S., Davis F., Higson S. P. J. (2012). Current trends in explosive detection techniques. *Talanta*.

[96] López-López M., García-Ruiz C. (2014). Infrared and Raman spectroscopy techniques applied to identification of explosives. *TRAC Trends in Analytical Chemistry*.

[97] Raza A., Biswas A., Zehra A., Mengesha A. (2020). Multiple tier detection of TNT using curcumin functionalized silver nanoparticles. *Forensic Science International: Synergy*.

[98] Wei W., Lu R., Tang S., Liu X. (2015). Highly cross-linked fluorescent poly(cyclotriphosphazene-co-curcumin) microspheres for the selective detection of picric acid in solution phase. *Journal of Materials Chemistry*.

[99] Akhavan J. (2004). Classification of explosive materials. *The Chemistry of Explosives*.

[100] Zhou X.-H., Li L., Li H.-H., Li A., Yang T., Huang W. (2013). A flexible Eu(iii)-based metal-organic framework: turn-off luminescent sensor for the detection of Fe(iii) and picric acid. *Dalton Transactions*.

[101] Wyman J. F., Serve M. P., Hobson D. W., Lee L. H., Uddin D. E. (1992). Acute toxicity, distribution, and metabolism of 2,4,6‐trinitrophenol (picric acid) in Fischer 344 rats. *Journal of Toxicology and Environmental Health*.

[102] Xiao J.-D., Qiu L.-G., Ke F. (2013). Rapid synthesis of nanoscale terbium-based metal-organic frameworks by a combined ultrasound-vapour phase diffusion method for highly selective sensing of picric acid. *Journal of Materials Chemistry*.

[103] Tu R., Liu B., Wang Z. (2008). Amine-capped ZnS−Mn2+ nanocrystals for fluorescence detection of trace TNT explosive. *Analytical Chemistry*.

[104] Freeman R., Finder T., Bahshi L., Gill R., Willner I. (2012). Functionalized CdSe/ZnS QDs for the detection of nitroaromatic or RDX explosives. *Advanced Materials*.

[105] Gao D., Wang Z., Liu B., Ni L., Wu M., Zhang Z.

[106] Gogoi B., Sen Sarma N. (2015). Curcumin-cysteine and curcumin-tryptophan conjugate as fluorescence turn on sensors for picric acid in aqueous media. *ACS Applied Materials & Interfaces*.

[107] Chakravarty S., Gogoi B., Sen Sarma N. (2015). Fluorescent probes for detection of picric acid explosive: a greener approach. *Journal of Luminescence*.

[108] Chakraborty S., Ilagan A. P. D. (2020). Chitosan curcumin film as a sensor for detection of o-nitrophenol and fluoride ion using fluoresce quenching technique. *Philippine Journal of Science*.

[109] Musilová J., Barek J., Pecková K. (2011). Determination of nitrophenols in drinking and river water by differential pulse voltammetry at boron-doped diamond film electrode. *Electroanalysis*.

[110] Pham T.-H., Lee B.-K., Kim J. (2016). Improved adsorption properties of a nano zeolite adsorbent toward toxic nitrophenols. *Process Safety and Environmental Protection*.

[111] Liu Y., Cai Y., Jiang X., Wu J., Le X. (2016). Molecular interactions, characterization and antimicrobial activity of curcumin-chitosan blend films. *Food Hydrocolloids*.

[112] Kumar M. K., Jha N. S., Yadav R., Jha S. K. (2019). Elucidation of confined copper nanospheres within self-assembled curcumin-cysteine and their electrocatalytic application. *Journal of the Electrochemical Society*.

[113] Golabi S. M., Zare H. R. (1999). Electrocatalytic oxidation of hydrazine at a chlorogenic acid (CGA) modified glassy carbon electrode. *Journal of Electroanalytical Chemistry*.

[114] Ensafi A. A., Mirmomtaz E. (2005). Electrocatalytic oxidation of hydrazine with pyrogallol red as a mediator on glassy carbon electrode. *Journal of Electroanalytical Chemistry*.

[115] Vernot E., MacEwen J. D., Bruner R. H. (1985). Long-term inhalation toxicity of hydrazine*∗*1. *Fundamental and Applied Toxicology*.

[116] Stone C. A., Dunn K. (1996). Vinylpyridine: an unusual cause of a chemical burn. *Burns*.

[117] Gogoi B., Sarma N. S. (2015). Poly-glycerol acrylate and curcumin composite: its dual emission fluorescence quenching and electrical properties for sensing 2-vinyl pyridine. *Journal of Materials Science*.

[118] Park S., Lee S.-Y. (2015). Significant enhancement of curcumin photoluminescence by a photosensitizing organogel: an optical sensor for pyrrole detection. *Sensors and Actuators B: Chemical*.

[119] Amshel C. E., Fealk M. H., Phillips B. J., Caruso D. M. (2000). Anhydrous ammonia burns case report and review of the literature. *Burns*.

[120] Ballal S. G., Ali B. A., Albar A. A., Ahmed H. O., Al-Hasan A. Y. (1998). Bronchial asthma in two chemical fertilizer producing factories in eastern Saudi Arabia. *International Journal of Tuberculosis & Lung Disease: The Official Journal of the International Union Against Tuberculosis and Lung Disease*.

[121] O’kane G. J. (1983). Inhalation of ammonia vapour. *Anaesthesia*.

[122] Choudat D., Goehen M., Korobaeff M., Boulet A., Dewitte J. D., Martin M. H. (1994). Respiratory symptoms and bronchial reactivity among pig and dairy farmers. *Scandinavian Journal of Work, Environment & Health*.

[123] Cormier Y., Israel-Assayag E., Racine G., Duchaine C. (2000). Farming practices and the respiratory health risks of swine confinement buildings. *European Respiratory Journal*.

[124] Walton M. (1973). Industrial ammonia gassing. *Occupational and Environmental Medicine*.

[125] Kim M., Lee H., Kim M., Park Y. C. (2021). Coloration and chromatic sensing behavior of electrospun cellulose fibers with curcumin. *Nanomaterials*.

[126] Sun M., Yu H., Zhu H. (2014). Oxidative cleavage-based near-infrared fluorescent probe for hypochlorous acid detection and myeloperoxidase activity evaluation. *Analytical Chemistry*.

[127] Pattison D. I., Davies M. J. (2001). Absolute rate constants for the reaction of hypochlorous acid with protein side chains and peptide bonds. *Chemical Research in Toxicology*.

[128] Pattison D. I., Davies M. J. (2006). Evidence for rapid inter- and intramolecular chlorine transfer reactions of histamine and carnosine chloramines: implications for the prevention of hypochlorous-acid-mediated damage. *Biochemistry*.

[129] Yue Y., Yin C., Huo F., Chao J., Zhang Y. (2014). The application of natural drug-curcumin in the detection hypochlorous acid of real sample and its bioimaging. *Sensors and Actuators B: Chemical*.

